# YTHDF2 promotes temozolomide resistance in glioblastoma by activation of the Akt and NF‐κB signalling pathways via inhibiting EPHB3 and TNFAIP3

**DOI:** 10.1002/cti2.1393

**Published:** 2022-05-09

**Authors:** Yu Chen, Yan‐Lan Wang, Kai Qiu, Yi‐Qiang Cao, Feng‐Jiang Zhang, Hai‐Biao Zhao, Xian‐Zhi Liu

**Affiliations:** ^1^ Department of Neurosurgery The First Affiliated Hospital of Zhengzhou University Zhengzhou China; ^2^ Department of Clinical Laboratory The Second Xiangya Hospital of Central South University Changsha China; ^3^ Department of Neurosurgery The First Affiliated Hospital of Kunming Medical University Kunming China

**Keywords:** EPHB3, GBM, TMZ, TNFAIP3, YTHDF2

## Abstract

**Objectives:**

Temozolomide (TMZ) resistance is a key factor that restricts the therapeutic effect of glioblastoma (GBM). YTH‐domain family member 2 (YTHDF2) is highly expressed in GBM tissues, while the mechanism of YTHDF2 in TMZ resistance in GBM remains not fully elucidated.

**Methods:**

The YTHDF2 expression in TMZ‐resistant tissues and cells was detected. Kaplan–Meier analysis was employed to evaluate the prognostic value of YTHDF2 in GBM. Effect of YTHDF2 in TMZ resistance in GBM was explored via corresponding experiments. RNA sequence, FISH in conjugation with fluorescent immunostaining, RNA immunoprecipitation, dual‐luciferase reporter gene and immunofluorescence were applied to investigate the mechanism of YTHDF2 that boosted TMZ resistance in GBM.

**Results:**

*YTHDF2* was up‐regulated in TMZ‐resistant tissues and cells, and patients with high expression of YTHDF2 showed lower survival rate than the patients with low expression of YTHDF2. The elevated YTHDF2 expression boosted TMZ resistance in GBM cells, and the decreased YTHDF2 expression enhanced TMZ sensitivity in TMZ‐resistant GBM cells. Mechanically, YTHDF2 bound to the N6‐methyladenosine (m^6^A) sites in the 3′UTR of EPHB3 and TNFAIP3 to decrease the mRNA stability. YTHDF2 activated the PI3K/Akt and NF‐κB signals through inhibiting expression of EPHB3 and TNFAIP3, and the inhibition of the two pathways attenuated YTHDF2‐mediated TMZ resistance.

**Conclusion:**

YTHDF2 enhanced TMZ resistance in GBM by activation of the PI3K/Akt and NF‐κB signalling pathways via inhibition of EPHB3 and TNFAIP3.

## Graphical abstract

In this study, we found that the elevated YTHDF2 expression induced by temozolomide stress decreased expression of EPHB3 and TNFAIP3 via inhibiting the mRNA stability in an m^6^A manner. Down‐regulation of EPHB3 and TNFAIP3 activated the PI3K/Akt and NF‐κB signaling pathway, enhanced proliferation and inhibited apoptosis of glioblastoma cells, and eventually enhanced the TMZ resistance in glioblastoma.

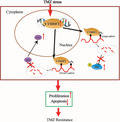

## Introduction

Glioblastoma (GBM) is an aggressive malignancy with high recurrence rate and fatality rate.^1^, ^2^ Although some progress has been made in clinical, the prognosis of GBM is still poor.^3^, ^4^ Temozolomide (TMZ) is a new generation of oral alkylating agents that cross the blood–brain barrier without side effects, which is widely applied in the GBM treatment.^5^ However, the emergence of TMZ resistance seriously restricts the clinical treatment of GBM.^6^, ^7^ Thus, exploring the novel molecules to enhance the TMZ resistance in GBM is conducive to ameliorate GBM.

Recently, the critical regulatory function of N6‐methyladenosine (m^6^A) RNA methylation in tumors has attracted wide attention.^8^ The m^6^A modification affects gene level by regulating RNA processing, localisation, translation, and the decay, which were regulated by m^6^A methyltransferase ‘writers’, m^6^A demethylase ‘erasers’ and m^6^A‐binding protein ‘readers’.^9^, ^10^, ^11^ Previous studies have confirmed that ‘writers’, ‘erasers’ and ‘readers’ are relevant to the GBM development.^12^, ^13^, ^14^ In the preliminary work, we discovered that the YTHDF2 level was elevated in TMZ‐resistant tissues and cells. Thus, we selected YTHDF2 for the follow‐up research. Furthermore, YTHDF2 is the first protein identified to be associated with m^6^A.^15^ YTHDF2 is mainly distributed in the cytoplasm and selectively recognises the m^6^A sites on the mRNA via its C‐terminal YTH domain to affect the mRNA degradation.^11^, ^15^ Furthermore, YTHDF2 controls the recruitment of carbon catabolite repressor protein 4‐NOT complex through the interaction between N‐terminal region of YTHDF2 and Src homology domain of the CNOT1 subunit, thus facilitating the degradation of target mRNAs.^16^ A large number of researches have demonstrated that YTHDF2 plays a crucial role in many physiological functions, including haematopoiesis, stem cell biology, and inflammation, and also regulates the cancer progression, such as acute myeloid leukaemia (AML), and liver cancer.^17^, ^18^, ^19^ In addition, Wang *et al*. have proven that YTHDF2 expression is increased in GBM, implying that YTHDF2 might participate in the progression of GBM.^20^ Here, we further corroborated that the YTHDF2 overexpression facilitated TMZ resistance in GBM cells. On the contrary, the interference with YTHDF2 elevated the sensitivity of TMZ‐resistant GBM cells to TMZ.

Earlier studies reported that the Akt/NF‐κB pathway was involved in the development of GBM resistance and could be mediated by various regulators. For example, Fujihara *et al*. have demonstrated that Ad‐DKK3 regulates the expression of MDR1 via the Akt/NF‐κB pathway and that promotes the anti‐tumor effect of TMZ in GBM cells.^21^ Tanaka *et al*. has confirmed that epidermal growth factor receptor mutation (EGFRvIII)‐activated mTORC2 signalling promotes GBM proliferation and chemotherapy resistance through the Akt‐independent activation of the NF‐κB pathway.^22^ In addition, Li *et al*. have claimed that YTHDF2 mediates the mRNA degradation of the tumor suppressor LHPP to induce AKT phosphorylation in prostate cancer.^23^ Chai *et al*. have reported that YTHDF2 facilitates UBXN1 mRNA degradation by recognising METTL3‐mediated m6A modification to activate the NF‐κB and exacerbates GBM.^24^ Based on this evidence, we speculated that the Akt/NF‐κB pathway was mediated by YTHDF2 in GBM cells.

In this work, we evaluated the YTHDF2 level in TMZ‐resistant tissues and cells in GBM and assessed the correlation between YTHDF2 expression and GBM resistance and prognosis. Moreover, we also explored the possible mechanism by which YTHDF2 mediates TMZ resistance in GBM.

## Results

### The elevated level of YTHDF2 is associated with the resistance of GBM and poor prognosis

**Figure 1:**
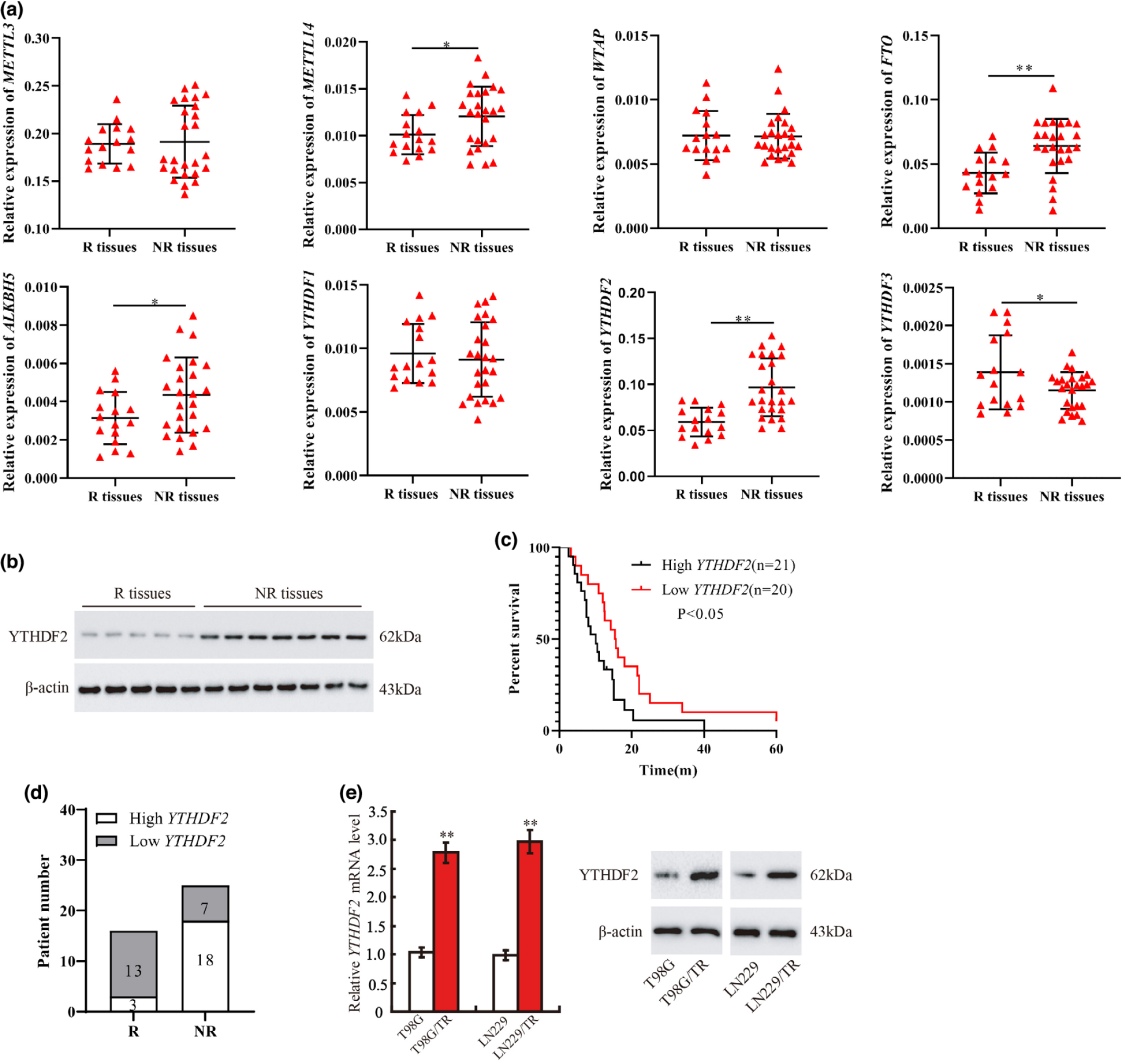
The elevated level of YTHDF2 is associated with the resistance of GBM and poor prognosis. The responder (R) GBM tissues (*n* = 16) and non‐responder (NR) GBM tissues (*n* = 25) were collected. **(a)** The expressions of m^6^A methyltransferases (*METTL3, METTL14, WTAP*), m^6^A demethylases (*FTO, ALKBH5*) and m^6^A‐binding protein (*YTHDF1, YTHDF2, YTHDF3*) were qualified by qRT‐PCR. **(b)** The protein level of YTHDF2 was measured by Western blot (R, *n* = 5; NR, *n* = 7). **(c)** Kaplan–Meier analysis of the survival rate in GBM patients with high expression of YTHDF2 (*n* = 21) or low expression of YTHDF2 (*n* = 20). **(d)** Patient number of YTHDF2 high expression or low expression in R (*n* = 16) and NR (*n* = 25) group. **(e)** Detection of YTHDF2 mRNA and protein level in T98G, T98G/TR, LN229 and LN229/TR cells (*n* = 3). *P < 0.05 vs. R tissues. **P < 0.01 vs. R tissues or T98G or LN229. GBM, glioblastoma.

The responder (R) GBM tissues and non‐responder (NR) GBM tissues were obtained. The expressions of m^6^A methyltransferase *METTL14*, m^6^A demethylase *FTO* and *ALKBH5* and m^6^A‐binding protein *YTHDF2* were increased in NR tissues, among which *YTHDF2* increased the most, while the expressions of m^6^A methyltransferase *METTL3*, *WTAP* and the m^6^A‐binding proteins *YTHDF1* had no significant change (Figure 1a). In contrast to these results, the YTHDF3 expression in NR tissues was reduced. Also, the fold change of YTHDF2 was more significant than YTHDF3 in NR tissues than in R tissues. Thus, we selected YTHDF2 in this research. The further results corroborated that the protein level of YTHDF2 was augmented in NR tissues (Figure 1b). Kaplan–Meier analysis of the overall survival time in GBM patients demonstrated that the survival rate of the patients with high level of *YTHDF2* was lower than that of the patients with *YTHDF2* low level (Figure 1c), and the high expression of YTHDF2 was positively correlated with the chemotherapy resistance (Supplementary table 2 & Figure 1d). Furthermore, YTHDF2 mRNA and protein levels were elevated in T98G/TR and LN229/TR cells (Figure 1e). Overall, the above data revealed that the elevated YTHDF2 level was related to the resistance of GBM and poor prognosis.

### Effect of YTHDF2 expression on TMZ resistance in GBM cells

**Figure 2:**
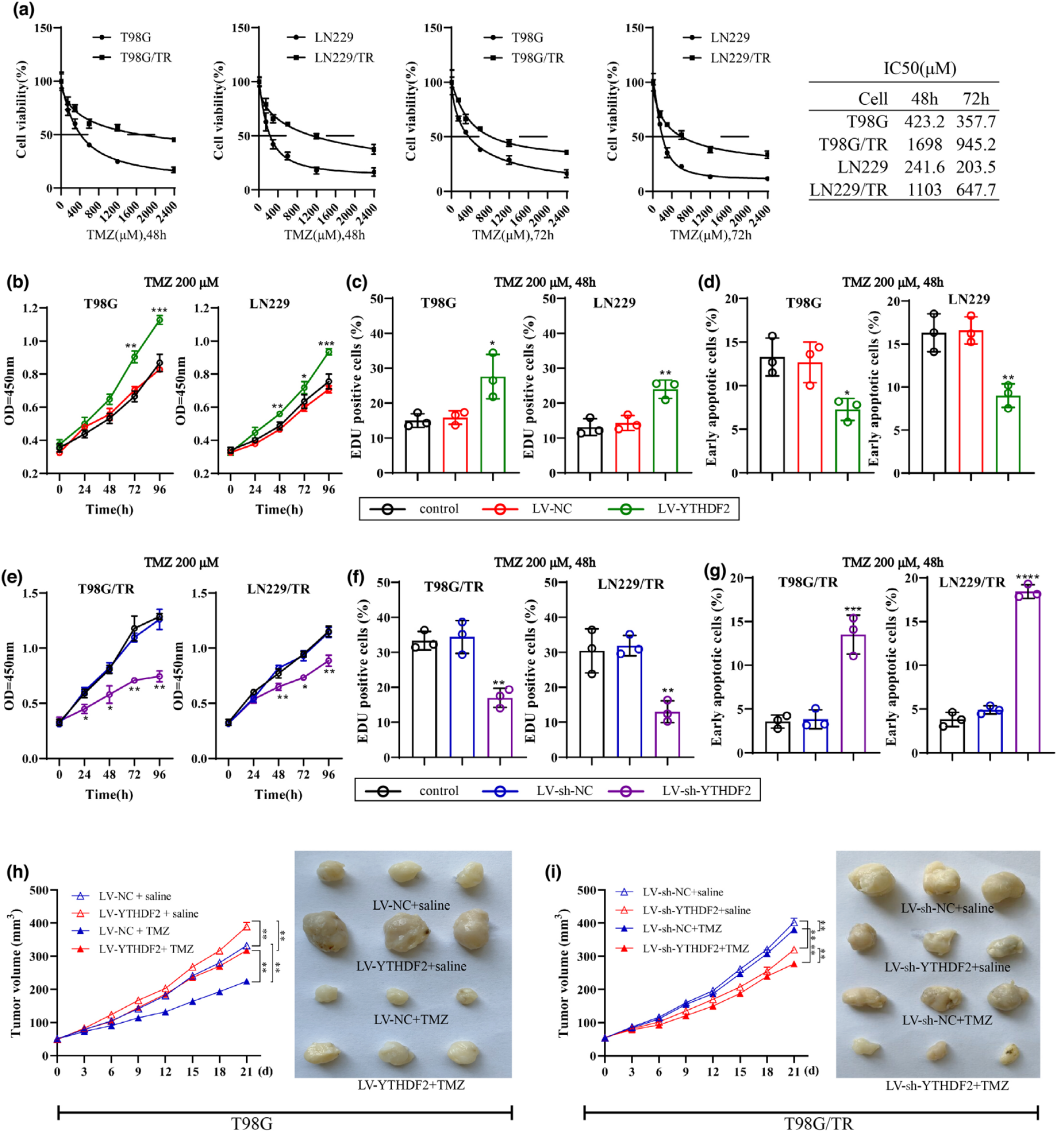
Effect of YTHDF2 on TMZ resistance in GBM cells. **(a)** The T98G and LN229 cells were treated with TMZ at different concentrations of 0, 150, 300, 600, 1200 and 2400 μM for 48 or 72 h. The IC50 value in each group was obtained. **(b)** The T98G and LN229 cells were infected with LV‐YTHDF2 or LV‐NC followed by 200 μm TMZ treatment for 0, 24, 48, 72 and 96 h. A CCK8 assay was performed to assess cell viability. **(c, d)** After infected with LV‐YTHDF2 or LV‐NC, the T98G and LN229 cells were treated with TMZ at 200 μm for 48 h. The cell proliferation and apoptosis were evaluated by an EdU assay and flow cytometry assay. **(e)** The T98G/TR and LN229/TR cells were infected with LV‐sh‐YTHDF2 or LV‐sh‐NC followed by 200 μm TMZ treatment for 0, 24, 48, 72 and 96 h. The cell viability was measured by the CCK8 assay. **(f, g)** The T98G/TR and LN229/TR cells infected with LV‐sh‐YTHDF2 or LV‐sh‐NC were treated with 200 μm TMZ for 48 h. The cell proliferation and apoptosis were detected. **(h, i)** T98G cells were infected with LV‐YTHDF2 or LV‐NC, and T98G/TR cells were infected with LV‐sh‐YTHDF2 or LV‐sh‐NC. Cells were then injected subcutaneously into the flanks of nude mice, and the mice were subsequently treated with 25 mg kg^−1^ TMZ by intraperitoneal injection every 2 days for 21 days. The intraperitoneal injection of saline was performed on the mice that had no TMZ injection. The experimental mice were grouped as follows (*n* = 7, per group): LV‐NC + saline, LV‐YTHDF + saline, LV‐NC + TMZ and LV‐YTHDF + TMZ; or LV‐sh‐NC + saline, LV‐sh‐YTHDF2 + saline, LV‐sh‐NC + TMZ and LV‐sh‐YTHDF2 + TMZ. After TMZ injection, the tumor size was measured every 2 days for 21 days. At day 21, the tumors were collected and photographed. The cell data are from three independent experiments. *P < 0.05, **P < 0.01, ***P < 0.001, ****P < 0.0001 vs. control or LV‐NC or LV‐sh‐NC. NC, negative control; TMZ, temozolomide.

We next detected the IC50 of TMZ for GBM cells (T98G, T98G/TR, LN229 and LN229/TR). TMZ treatment was performed at different concentrations of 0, 150, 300, 600, 1200 and 2400 μM for 48 h or 72 h. The result showed that the lowest IC50 value was nearly 200 μm (Figure 2a); thus, the concentration of 200 μm was selected for the following experiment. To understand the potential effect of YTHDF2 on the TMZ resistance in GBM cells or the TMZ sensitivity of GBM resistant cells, LV‐YTHDF2 was infected into T98G and LN229 cells to enhance YTHDF2 expression, and LV‐sh‐YTHDF2 was infected into T98G/TR and LN229/TR cells to silence YTHDF2. The efficiency of infection was confirmed by Western blot (Supplementary figure 1a). After infection of LV‐YTHDF2, cells were subjected to TMZ for different times. A CCK8 assay was conducted to evaluate the viability of T98G and LN229 cells, and the result revealed a significant increase of cell viability in LV‐YTHDF2 infection group (Figure 2b). Similarly, in comparison with control group and LV‐NC infection group, the number of EdU‐positive cells was markedly enhanced in LV‐YTHDF2 infection group, implying the increase of cell proliferation (Figure 2c & Supplementary figure 1c). Meanwhile, we discovered that LV‐YTHDF2 infection remarkably inhibited apoptosis of T98G and LN229 cells (Figure 2d). In addition to these results, the subsequently results conveyed that YTHDF2 silencing significantly reduced the proliferation of T98G/TR and LN229/TR cells, while increased cell apoptosis (Figure 2e–g & Supplementary figure 1d–f). In the *in vivo* experiment, T98G cells infected with LV‐YTHDF2 were transplanted into mice followed by TMZ subjection. As indicated, the overexpression of YTHDF2 promoted the tumor growth, while TMZ treatment abrogated the effect (Figure 2h & Supplementary figure 1b). In addition, T98G/TR or LN229/TR cells infected with LV‐sh‐YTHDF2 were transplanted into mice followed by TMZ treatment. Notably, the tumor growth was inhibited in LV‐sh‐YTHDF2 infection group, and TMZ treatment further exacerbated this inhibitory effect (Figure 2i & Supplementary figures1b, 3i). Collectively, these data suggested that the YTHDF2 overexpression conferred TMZ resistance, promoted proliferation and restrained apoptosis of GBM cells, while YTHDF2 silencing exerted the adverse effects.

### YTHDF2 decreased the mRNA stability of EPHB3 in an m^6^A manner

Previous studies have demonstrated that YTHDF2 promotes the mRNA degradation of genes in various diseases.^25^, ^26^ To further investigate the downstream targets of YTHDF2, RNA sequence and RIP‐sequence analyses were performed using GSE158742 database based on YTHDF2 silencing in GBM stem cell samples from GBM patients. According to the result, we obtained 47 mRNAs that were up‐regulated (logFC > 0 & *P* < 0.05) after silencing of YTHDF2 and significantly enriched (logFC > 0 & *P* < 0.05) after adding the anti‐YTHDF2 (Supplementary figure 2a). Then, the 47 genes were detected in T98G/TR and LN229/TR cells after infection of LV‐Sh‐YTHDF2. The common up‐regulated genes were displayed in Supplementary figure 2b. By using the RIP assay, KIAA1549L, EPHB3, TNFRSF1B, BAALC, TAOK3, BAMB1 and TNFAIP3 were confirmed to interact with YTHDF2, in which EPHB3 and TNFAIP3 interact with YTHDF2 more significant (Supplementary figure 2c). In addition, EPHB3 and TNFAIP3 had the anti‐tumor effects. The further result revealed that another shRNA against YTHDF2 markedly elevated the mRNA and protein level of EPHB3 and TNFAIP3 in T98G/TR cells (Supplementary figure 3a, b). We therefore focused on EPHB3 and TNFAIP3 in the following experiments.

**Figure 3:**
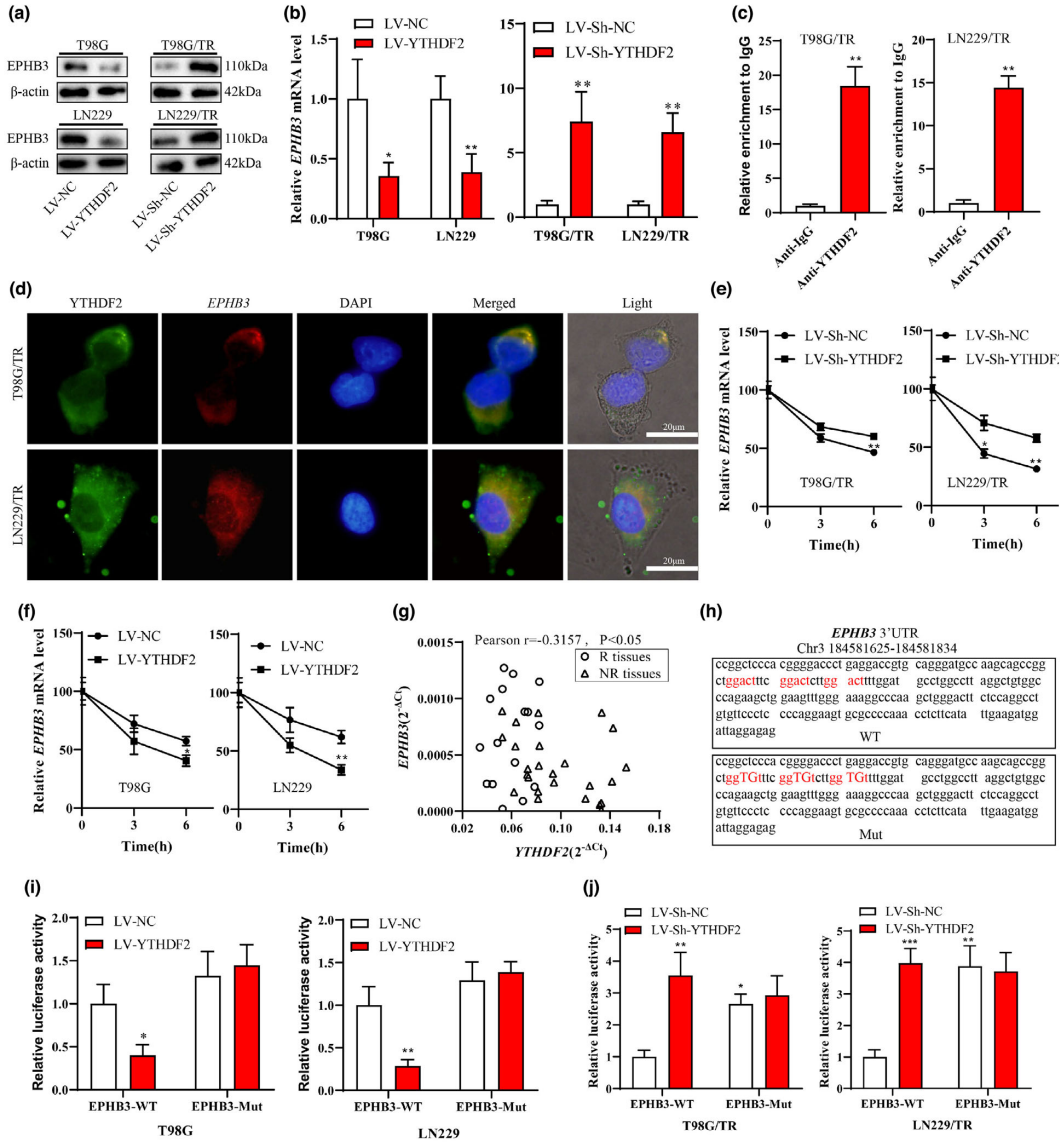
YTHDF2 decreased the mRNA stability of EPHB3 in an m^6^A manner. **(a, b)** The LV‐YTHDF2 or LV‐NC was infected into T98G and LN229 cells, and the LV‐sh‐YTHDF2 or LV‐sh‐NC was infected into T98G/TR and LN229/TR cells. The protein and mRNA level of EPHB3 was measured by Western blotting and qRT‐PCR. **(c)** RNA immunoprecipitation (RIP) was performed in T98G/TR and LN229/TR cells using anti‐YTHDF2 antibody. **(d)** FISH in conjugation with fluorescent immunostaining analysis was performed in T98G/TR and LN229/TR cells. **(e)** T98G/TR and LN229/TR cells were infected with LV‐sh‐YTHDF2 or LV‐sh‐NC followed by treatment of 5 μm Actinomycin‐D for 0, 3 and 6 h. The relative mRNA level of EPHB3 was measured. **(f)** T98G and LN229 cells infected with LV‐YTHDF2 or LV‐NC were treated with 5 μm Actinomycin‐D for 0, 3 and 6 h followed by the detection of EPHB3 expression. **(g)** Correlation analysis of YTHDF2 and PTEN expression in GBM tissues (*r* = −0.3157, *P* < 0.05). **(h)** The sequence of luciferase reporter gene vector EPHB3‐3′UTR (EPHB3‐WT) and EPHB3‐3′UTR with mutant m6A sites (EPHB3‐Mut) were established and exhibited. **(i, j)** A dual‐luciferase reporter gene assay was performed in T98G, LN229, LN229/TR, and T98G/TR cells to detect the interaction between YTHDF2 and EPHB3. Data are from three independent experiments.*P < 0.05, **P < 0.01 vs. LV‐sh‐NC or LV‐NC or anti‐IgG. Scale bar: 20 μm.

As previously described, EPHB3 could inhibit lung cancer cell migration and suppress PI3K/Akt signalling in GBM.^27^, ^28^ The decreased EPHB3 expression was correlated with the reduced survival rate of GBM patients, and blocking the PI3K/Akt/mTOR pathway sensitised GBM cells to apoptosis upon TMZ treatment.^29^ We next discovered that the overexpression of YTHDF2 decreased the protein and mRNA levels of EPHB3 in T98G and LN229 cells, and the knockdown of YTHDF2 enhanced these levels in T98G/TR and LN229/TR cells (Figure 3a, b). According to RIP experiment, we found that YTHDF2 could bind to the mRNA of *EPHB3* (Figure 3c). Next, we conducted a FISH assay to assess the co‐localisation of *EPHB3* mRNA and *YTHDF2* in T98G/TR and LN229/TR cells. As expected, the results confirmed that *YTHDF2* and *EPHB3* mRNA were co‐localised in the cytoplasm (Figure 3d). Subsequently, the LV‐sh‐YTHDF2 or LV‐sh‐NC was infected into T98G/TR and LN229/TR cells, and LV‐YTHDF2 or LV‐NC was infected into T98G and LN229 cells, followed by treatment of Actinomycin‐D for 3 or 6 h to inhibit the RNA synthesis. The data suggested that YTHDF2 silencing remarkably inhibited RNA degradation in GBM cells, while the YTHDF2 overexpression promoted the RNA degradation (Figure 3e, f). Subsequently, the Pearson correlation analysis indicated that the YTHDF2 level was negatively correlated to the EPHB3 level in GBM tissues (Figure 3g).

Recent studies have claimed that the YTHDF2 could bind to m6A site in 3′UTR of mRNA and accelerate the degradation of target gene mRNAs.^25^, ^30^ We established the luciferase reporter gene vectors of EPHB3‐3′UTR (EPHB3‐WT) and EPHB3‐3′UTR with mutant m6A sites (EPHB3‐Mut), and the sequences were displayed in Figure 3h. Furthermore, the infection of LV‐YTHDF2 in T98G and LN229 cells reduced the luciferase activity of EPHB3‐WT (with m^6^A sites), and the infection of LV‐sh‐YTHDF2 in T98G/TR cells and LN229/TR cells increased the luciferase activity of EPHB3‐WT. However, both the LV‐YTHDF2 and LV‐sh‐YTHDF2 infections exerted no significant influence on the luciferase activity of EPHB3‐Mut (with mutant m^6^A sites) (Figure 3i, j & Supplementary figure 3c). Additionally, the previous evidence has suggested that the K416/R527 site of YTHDF2 is crucial for RNA backbone binding, and the W432/W486/W491 sites of YTHDF2 are significant for m6A modification.^31^ We constructed the YTHDF2 (WT), mutants of YTHDF2‐2A‐Mut, YTHDF2‐3A‐Mut, and YTHDF2‐5A‐Mut, and transfected them into T98G and LN229 cells. The results showed that transfection of YTHDF2 (WT) down‐regulated EPHB3 expression, while the transfection of YTHDF2‐2A, YTHDF2‐3A or YTHDF2‐5A had no significant effect on EPHB3 level (Supplementary figure 2d). Overall, these data indicated that YTHDF2 decreased the mRNA stability of EPHB3 in an m^6^A manner.

### YTHDF2 activated the PI3K/Akt signalling pathway by restraining EPHB3 expression

**Figure 4:**
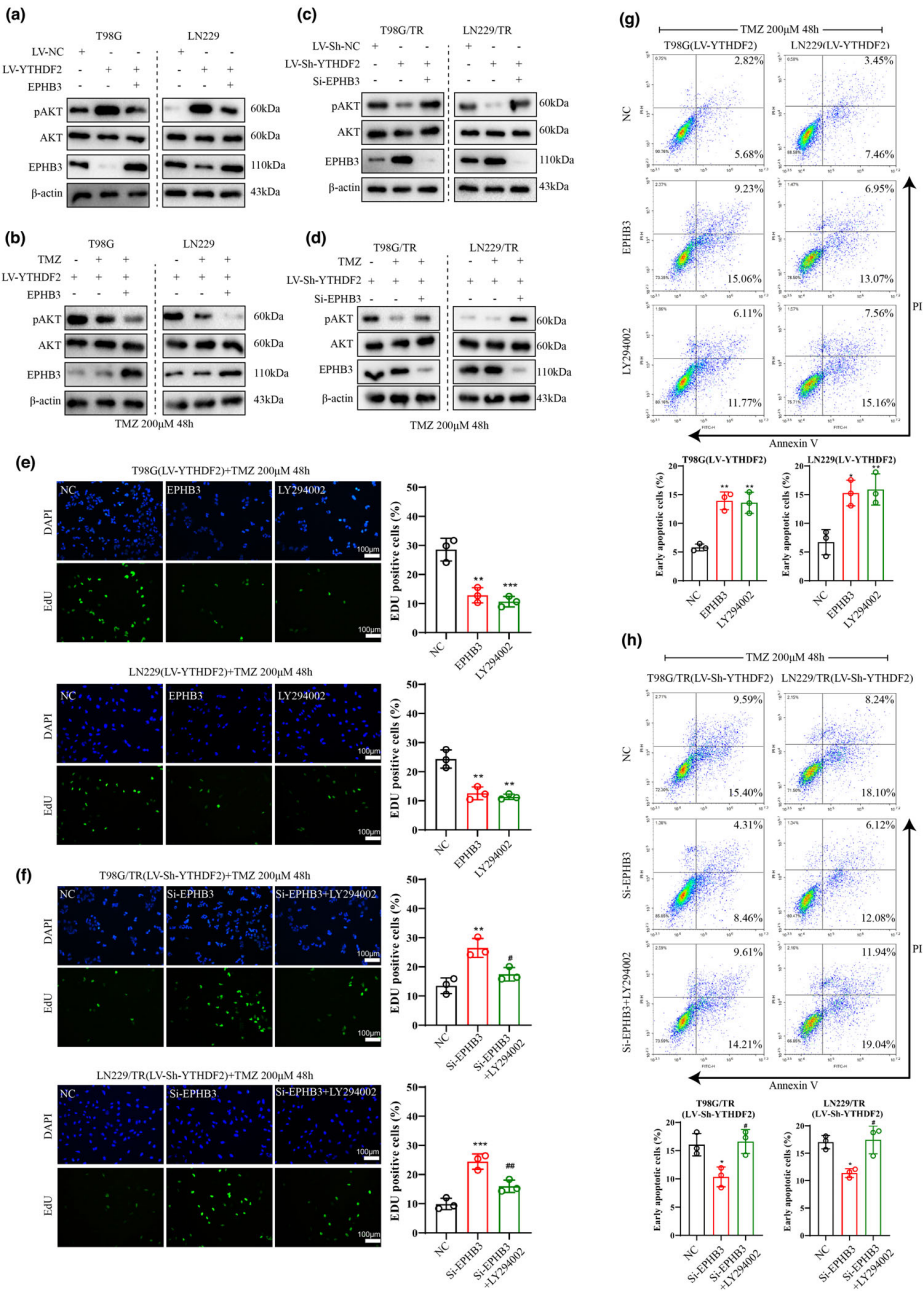
YTHDF2 activated PI3K/Akt signalling through inhibiting EPHB3 expression. **(a–d)** The LV‐YTHDF2 or LV‐NC was infected or co‐infected into T98G and LN229 cells with or without TMZ treatment (200 μM), and the LV‐sh‐YTHDF2 or LV‐sh‐NC was infected or co‐infected into T98G/TR and LN229/TR cells with or without TMZ treatment. The protein levels of Akt and EPHB3, and level of phosphorylated AKT were measured. **(e)** In T98G and LN229 cells, the LV‐YTHDF2 was co‐infected with LV‐EPHB3 or LV‐NC, or the PI3K inhibitor LY294002 (10 μm) was added and cultured for 24 h after LV‐YTHDF2 infection. Cells were then treated with 200 μM TMZ, and the percentage of EdU‐positive cells was calculated. **(f)** The LV‐sh‐YTHDF2 was co‐infected with LV‐sh‐EPHB3 or LV‐NC in T98G/TR and LN229/TR cells, or cells were infected with LV‐sh‐YTHDF2 and transfected with si‐EPHB3 followed by treatment of LY294002 and TMZ for 24 h. The percentage of EdU‐positive cells was analysed. **(g, h)** The LV‐YTHDF2 was co‐infected with LV‐EPHB3 or LV‐NC into T98G and LN229 cells, or the LV‐sh‐YTHDF2 was infected into T98G/TR and LN229/TR cells followed by si‐EPHB3 or si‐NC transfection with or without LY294002 treatment for 24 h. Then, all the cells were subjected to 200 μm TMZ treatment for 48 h. Data are from three independent experiments. *P < 0.05, **P < 0.01, ***P < 0.001 vs. NC; #*P* < 0.05, ##*P* < 0.01 vs. si‐EPHB3. NC: LV‐NC or si‐NC; Scale bar: 100 μm.

Recent studies have clarified that EPHB3 suppresses the activation of the PI3K/Akt signalling.^32^, ^33^ We sought to explore whether YTHDF2 regulates the PI3K/Akt signalling pathway via EPHB3. As shown in Figure 4a, b, the YTHDF2 overexpression elevated the level of phosphorylated AKT, but decreased EPHB3 level in T98G and LN229 cells with or without TMZ treatment, while these trends were reversed after the overexpression of EPHB3. In addition, the knockdown of YTHDF2 decreased the level of phosphorylated AKT, but increased the EPHB3 level in T98G/TR and LN229/TR cells with or without TMZ treatment, while these effects were partly abolished by the transfection of si‐EPHB3 (Figure 4c, d). We next explored whether YTHDF2 regulated cell activities through the EPHB3/PI3K/Akt axis. T98G and LN229 cells were infected with LV‐YTHDF2 and LV‐NC, or infected with LV‐YTHDF2 and LV‐EPHB3, or infected with LV‐YTHDF2 and treated with the PI3K inhibitor LY294002, followed by TMZ treatment. The data showed that the infection of LV‐YTHDF2 and LV‐EPHB3 markedly reduced the number of EdU‐positive cells, and the LV‐YTHDF2 infection and LY294002 treatment showed the similar results (Figure 4e). Furthermore, we found that the knockdown of EPHB3 enhanced the number of EdU‐positive cells under TMZ condition, while the LY294002 treatment reversed this effect (Figure 4f). According to the flow cytometry result, the overexpression of EPHB3 elevated apoptosis of T98G and LN229 cells under TMZ treatment, and LY294002 treatment showed similar results. However, the knockdown of EPHB3 reduced apoptosis of T98G/TR and LN229/TR cells under TMZ treatment (Figure 4g, h). These findings revealed that YTHDF2 activated the PI3K/Akt signalling pathway by repressing EPHB3 expression, and the inhibition of PI3K/Akt signalling attenuated YTHDF2‐mediated TMZ resistance.

### YTHDF2 inhibited the mRNA stability of TNFAIP3 in an m^6^A manner

**Figure 5:**
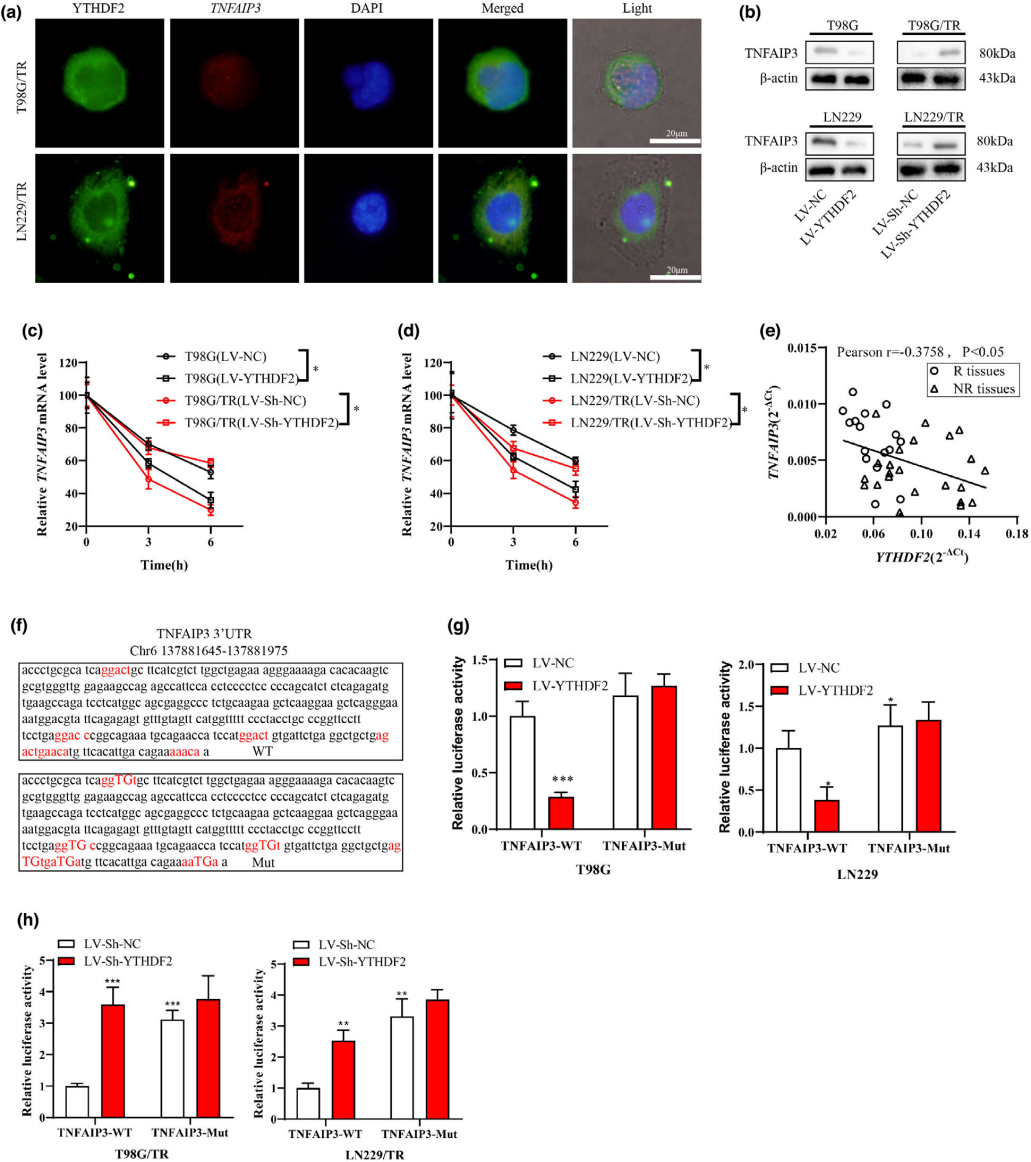
YTHDF2 inhibited the mRNA stability of TNFAIP3 in m6A manner. **(a)** FISH in conjugation with fluorescent immunostaining analysis was performed to confirm the co‐localisation in T98G/TR and LN229/TR cells. **(b)** The LV‐YTHDF2 or LV‐NC was infected into T98G and LN229 cells, and the LV‐sh‐YTHDF2 or LV‐sh‐NC was infected into T98G/TR and LN229/TR cells followed by the TNFAIP3 protein level detection. **(c, d)** T98G and LN229 cells infected with LV‐YTHDF2 or LV‐NC, and T98G/TR and LN229/TR cells infected with LV‐sh‐YTHDF2 or LV‐sh‐NC were treated with 5 μm Actinomycin‐D for 0, 3 and 6 h. The relative mRNA level of TNFAIP3 was detected to assess the RNA degradation. **(e)** Correlation analysis of the YTHDF2 level and TNFAIP3 level in GBM tissues (*r* = −0.3758, *P* < 0.05). **(f)** The sequences of luciferase reporter gene vector TNFAIP3‐3′UTR (TNFAIP3‐WT) and TNFAIP3‐3′UTR with mutant m6A sites (TNFAIP3‐Mut). **(g, h)** A dual‐luciferase reporter gene assay was performed in T98G, LN229, LN229/TR and T98G/TR cells to identify the interaction between YTHDF2 and TNFAIP3. Data are from three independent experiments. *P < 0.05, **P < 0.01, ***P < 0.001 vs. LV‐NC or LV‐sh‐NC; Scale bar: 20 μm.

To determine the interaction between YTHDF2 and TNFAIP3, we conducted a FISH assay to assess the co‐localisation of *TNFAIP3* mRNA and *YTHDF2* in T98G/TR and LN229/TR cells. As indicated in Figure 5a, *YTHDF2* and *TNFAIP3* mRNA were co‐localised in the cytoplasm. Then, LV‐YTHDF2 or LV‐NC was infected into T98G and LN229 cells, and LV‐Sh‐YTHDF2 or LV‐Sh‐NC was infected into T98G/TR and LN229/TR cells. It can be seen that the overexpression of YTHDF2 reduced the TNFAIP3 protein level, while the knockdown of YTHDF2 enhanced this level (Figure 5b). The actinomycin‐D was next added to inhibit the RNA synthesis in GBM cells. We discovered that the overexpression of YTHDF2 promoted RNA degradation, and the silence of YTHDF2 inhibited RNA degradation (Figure 5c, d). The Pearson correlation result indicated that the YTHDF2 level was negatively correlated to the TNFAIP3 level in GBM tissues (Figure 5e). We established the vectors of TNFAIP3‐3′UTR (TNFAIP3‐WT) and TNFAIP3‐3′UTR with mutant m^6^A site (TNFAIP3‐Mut), and the sequences were displayed in Figure 5f. The result suggested that the infection of LV‐YTHDF2 in T98G and LN229 cells reduced the luciferase activity of TNFAIP3‐WT (with m^6^A sites), and the infection of LV‐Sh‐YTHDF2 in T98G/TR and LN229/TR cells increased the luciferase activity of TNFAIP3‐WT. Whereas, both the LV‐YTHDF2 and LV‐sh‐YTHDF2 infection exerted no significant effect on the luciferase activity of TNFAIP3‐Mut (with mutant m^6^A sites) (Figure 5g, h). In conclusion, YTHDF2 reduced the mRNA stability of TNFAIP3 in an m6A manner.

### YTHDF2 activated the NF‐κB signalling through inhibiting TNFAIP3 expression

**Figure 6:**
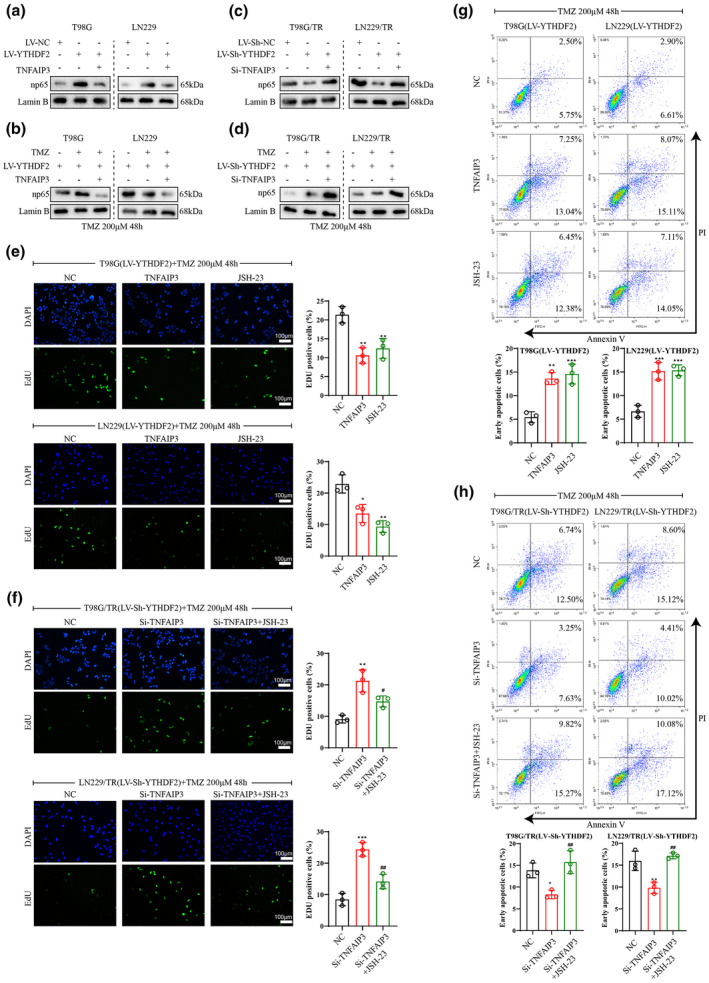
YTHDF2 activated NF‐κB signalling through inhibiting TNFAIP3 expression. **(a, b)** T98G and LN229 cells infected with LV‐YTHDF2 or LV‐NC, or co‐infected with LV‐YTHDF2 and LV‐TNFAIP3. The nuclear and cytoplasm was separated, and p65 protein level in the nuclear was measured. **(c, d)** T98G/TR and LN229/TR cells were infected with LV‐sh‐YTHDF2 or LV‐sh‐NC, or infected with LV‐sh‐YTHDF2 and transfected with si‐TNFAIP3, following by the nuclear p65 protein level detection. **(e)** The LV‐YTHDF2 was co‐infected with LV‐TNFAIP3 or LV‐NC in T98G and LN229 cells, or cells were infected with LV‐YTHDF2 and treated with JSH‐23 (NF‐κB inhibitor, 20 μm) for 24 h. Next, 200 μm TMZ was added to cells and cultured for 48 h. Cell proliferation was determined by an EdU assay. **(f)** LV‐sh‐YTHDF2 was infected into T98G/TR and LN229/TR cells followed by si‐TNFAIP3 or si‐NC transfection with or without treatment of JSH‐23 (NF‐κB inhibitor, 20 μm). After incubation with TMZ (200 μm) for 48 h, the percentage of EdU‐positive cells was calculated. **(g)** After co‐infection of LV‐YTHDF2 and LV‐NC or LV‐TNFAIP3, or treated with 20 μm JSH‐23 after infection of LV‐YTHDF2, the apoptosis of T98G and LN229 cells was evaluated by flow cytometry. **(h)** The apoptosis of T98G/TR and LN229/TR cells was assessed after infection of LV‐sh‐YTHDF2 and transfection of si‐NC or si‐TNFAIP3 with or without 20 μm JSH‐23 treatment. Data are from three independent experiments. *P < 0.05, **P < 0.01, ***P < 0.001 vs. NC; #*P* < 0.05 vs. si‐TNFAIP3; NC: LV‐NC or si‐NC; Scale bar: 100 μm.

After the infection or co‐infection of LV‐YTHDF2 and LV‐TNFAIP3 into T98G and LN229 cells with or without TMZ treatment, we observed that the infection of LV‐YTHDF2 enhanced expression level of p65 in nuclear, while the co‐infection of LV‐YTHDF2 and LV‐TNFAIP3 reduced this level (Figure 6a, b). Meanwhile, the YTHDF2 silencing reduced p65 level in the nuclear of T98G/TR and LN229/TR cells with or without TMZ treatment, while the silence of YTHDF2 and TNFAIP3 reversed this effect (Figure 6c, d). Next, T98G and LN229 cells were infected with LV‐YTHDF2 and LV‐NC, or infected with LV‐YTHDF2 and LV‐TNFAIP3, or infected with LV‐YTHDF2 and treated with NF‐κB inhibitor JSH‐23, followed by TMZ treatment. The EdU result displayed that either overexpression of YTHDF2 and TNFAIP3 or overexpression of YTHDF2 with JSH‐23 treatment reduced proliferation of T98G and LN229 cells (Figure 6e). In addition, we discovered that the knockdown of YTHDF2 and TNFAIP3 significantly induced proliferation of T98G/TR and LN229/TR cells with TMZ treatment, while the knockdown of YTHDF2 and TNFAIP3 with JSH‐23 treatment partly abolished the effect (Figure 6f). Based on the flow cytometry result, the overexpression of YTHDF2 and TNFAIP3 or the overexpression of YTHDF2 with JSH‐23 treatment increased apoptosis of T98G and LN229 cells under TMZ treatment, whereas the knockdown of YTHDF2 and TNFAIP3 reduced apoptosis of T98G/TR and LN229/TR cells, and the knockdown of YTHDF2 and TNFAIP3 with JSH‐23 treatment reversed this result (Figure 6g, h).

The lentivirus containing YTHDF2 was silenced by another shRNA (LV‐sh‐YTHDF2′) and was infected with T98G/TR cells followed by si‐EPHB3 or si‐TNFAIP3 transfection with or without LY294002 or JSH‐23 treatment. The result showed that, under TMZ treatment, knockdown of YTHDF2′ and EPHB3 increased the viability and proliferation of T98G/TR cells compared with YTHDF2′ knockdown alone, while the LY294002 treatment reduced this level. Similarly, the knockdown of YTHDF2′ and TNFAIP3 increased the viability and proliferation of T98G/TR cells compared with YTHDF2′ knockdown alone, whereas the JSH‐23 subjection decreased the level (Supplementary figure 3d, e). Differently, the apoptotic rate of T98G/TR cells showed the opposite result (Supplementary figure 3f). Under TMZ treatment, the LV‐sh‐YTHDF2′ transfection in T98G/TR cells reduced the level of phosphorylated AKT and increased EPHB3 level, while co‐transfection of LV‐sh‐YTHDF2′ and si‐EPHB3 reversed this effect (Supplementary figure 3g). Likely, the LV‐sh‐YTHDF2′ transfection in T98G/TR cells reduced the nuclear level of p65 and increased the TNFAIP3 level, while co‐transfection of LV‐sh‐YTHDF2’ and si‐TNFAIP3 reversed this level (Supplementary figure 3h). In addition, the knockdown of YTHDF2 and EPHB3 or the knockdown of YTHDF2 and TNFAIP3 promoted the tumor growth in xenograft model under TMZ treatment compared with YTHDF2 silence alone (Supplementary figure 3j). Taken together, these data suggested that YTHDF2 activated NF‐κB signalling through inhibiting TNFAIP3 expression, and the inhibition of NF‐κB signalling attenuated YTHDF2‐mediated TMZ resistance.

## Discussion

This work is designed to comprehend the potential mechanism of YTHDF2 in TMZ resistance in GBM. Herein, we confirmed that the YTHDF2 level was elevated in TMZ‐resistant tissues and cells in GBM, which was consistent with the previous description.^20^ Recently, a study by Dixit *et al*. has demonstrated that the YTHDF2 is preferentially expressed in GBM stem cells.^34^ Our studies further demonstrated that the silence of YTHDF2 increased the sensitivity of TMZ‐resistant GBM cells. For the exploration of the regulatory mechanisms, we found that YTHDF2 repressed the mRNA stability of EPHB3 or TNFAIP3 in an m^6^A manner, implying that the YTHDF2/EPHB3/TNFAIP3 may be a novel regulation axis in TMZ resistance in GBM. Furthermore, the down‐regulation of EPHB3 or TNFAIP3 activated the PI3K/Akt and NF‐κB signalling pathways, leading to the promotion of GBM cell proliferation and the repression of cell apoptosis, eventually facilitated the TMZ resistance in GBM cells.

Accumulating evidence indicates that the gradual emergence of TMZ resistance is the main reason for the failure of TMZ in the GBM treatment.^35^, ^36^ Thus, the key molecules and potential signalling pathways that facilitated the TMZ resistance in GBM was determined in the following. Emerging evidence demonstrates that m^6^A modification is a momentous mRNA modification in GBM, which is closely associated with the activation and inhibition of cancer pathways.^37^ So far, studies have found that the regulation of mRNA m^6^A level affects several aspects of GBM, including growth, chemoresistance and tumorigenesis, suggesting that the modification of mRNA m^6^A might be a promising target for GBM therapy.^8^, ^38^ YTHDF2 is an m^6^A‐binding protein and can recognise the m^6^A site in the 3’UTR of mRNAs.^39^ What arouses our concern is that YTHDF2 is highly expressed in GBM tissues.^8^ Likely, our work also corroborated the up‐regulation of YTHDF2 in TMZ‐resistant tissues, and we further confirmed that the decreased YTHDF2 expression enhanced the sensitivity of GBM TMZ‐resistant cells. Based on this novel discovery, targeting YTHDF2 may be a valid strategy for the treatment of GBM.

Both the EPHB3 and TNFAIP3 are the typical tumor suppressors.^40^, ^41^ The aberrant expressions of EPHB3 and TNFAIP3 are relevant to the progression of GBM.^42^, ^43^ In our experiments, we performed RNA sequence on T98G/TR and LN229/TR cells after the knockdown of YTHDF2, and the result corroborated that EPHB3 and TNFAIP3 expression were up‐regulated. Importantly, the qRT‐PCR and Western blot detection showed the similar results, implying a possible interaction between YTHDF2 and EPHB3 or TNFAIP3. Our further results showed that YTHDF2 could bind to the 3'UTR of EPHB3 or TNFAIP3 with m^6^A sites. Previous evidence indicated that YTHDF2 was involved in the regulation of mRNA stability.^11^ Similarly, we confirmed that YTHDF2 decreased the mRNA stability of EPHB3 and TNFAIP3 in an m^6^A manner.

The abnormal activation of some signalling pathways is crucial in regulating TMZ resistance. For instance, blocking the PI3K/Akt signalling enhances GBM cell sensitivity to TMZ.^44^, ^45^ Notably, EPHB3 has been confirmed to regulate the PI3K/Akt signalling pathway in the progression of various tumors.^46^ TNFAIP3 encodes the NF‐κB regulatory protein A20 and mediates the suppression of NF‐κB,^47^ and the decreased TNFAIP3 expression in GBM is closely related to TMZ resistance.^42^, ^48^ Previous studies demonstrated that YTHDF2 regulated the Akt or NF‐κB pathways in prostate cancer or GBM through facilitating relevant gene mRNA degradation.^23^, ^24^ Combined with the previous findings that YTHDF2 repressed the mRNA stability of EPHB3 or TNFAIP3 in an m^6^A manner, we further corroborated that YTHDF2 activated the PI3K/Akt and NF‐κB signalling pathways through decreasing expression of EPHB3 and TNFAIP3. Moreover, studies have suggested that activation of the NF‐κB signalling pathway enhances the resistance of GBM cells to TMZ.^49^, ^50^ These data could support our data to some extent.

Taken together, we confirm the YTHDF2 function in conferring TMZ resistance by decreasing the mRNA stability of EPHB3 or TNFAIP3 in an m^6^A manner. Furthermore, we identify that YTHDF2 facilitates TMZ resistance in GBM by the activation of the PI3K/Akt and NF‐κB signalling pathways through inhibiting expression of EPHB3 and TNFAIP3. Our study may provide novel insights for enhancing the TMZ resistance in GBM, which is of great significance for ameliorating GBM.

## Methods

### Human samples

All the responder (R) GBM tissues (*n* = 16) and non‐responder (NR) GBM tissues (*n* = 25) were collected from the patients with primary GBM after obtaining the informed consent from the enrolled patients. The patients were classified according to the WHO and RECIT criteria for sensitivity of solid tumors to chemotherapy. CR (complete remission): the tumor disappeared completely and remained for more than 4 weeks; PR (partial remission): the product of the maximum diameter and vertical diameter of the tumor was reduced more than 50% and remained for more than 4 weeks, without the formation of new lesions; SD (stable disease): the two‐path product of lesions decreased less than 50%, or increased less than 25%, and remained for more than 4 weeks without the emergence of new lesions; PD (progressive disease): the product of two diametral lesions increased more than 25% or appeared new lesions. The whole samples were treated by TMZ (dosage of 150–200 mg per m^2^ per day), and four weeks was a period of treatment. Tumor response status was assessed referring to the WHO and Response Evaluation Criteria in Solid Tumors (RECIST) version 1.0 criteria. This study was approved by the Ethics Committee of the First Affiliated Hospital of Zhengzhou University (2019‐KY‐176).

### Quantitative real‐time PCR

The GBM tissues or GBM cells were subjected with Trizol Reagent (Invitrogen, Carlsbad, CA, USA) to isolate total RNAs. The quality of the total RNAs was examined using absorbance at 260 and 280 nm. Then, the cDNA was synthesised with the Primer ScriptTM RT reagent kit (TaKaRa, Japan). The qRT‐PCR was performed on a 7500 Fast Real‐Time PCR System (Applied Biosystems, USA) with the SYBR Master Mix (Takara). β‐actin was employed as the endogenous control. The 2^−ΔΔ^ *^C^* ^t^ method was adopted to calculate gene expressions. The primer sequences are shown in Supplementary table 1.

### Western blots

The GBM tissues or GBM cells were added with RIPA buffer (Beyotime, Shanghai, China) containing protease inhibitors. The concentration of aboveextracted proteins was assessed with a BCA assay kit (Abcam, Cambridge, UK). The equal amount of protein samples (30 μg) was separated by 10% SDS‐PAGE and then transferred into the PVDF membranes (Invitrogen). The primary antibodies including anti‐YTHDF2 (ab220163, Abcam), anti‐EPHB3 (ab133742, Abcam), anti‐TNFAIP3 (ab92324, Abcam), anti‐pAkt (Ser473, #3787, Cell Signaling technology, Bossdun, USA), anti‐Akt (#2938, Cell Signaling), anti‐p65 (ab32536, Abcam), anti‐LaminB (sc‐374015, Santa Cruz Biotechnology, CA, USA) and β‐actin (ab8227, Abcam) were incubated with the PVDF membranes for overnight at 4°C. The samples were next incubated with the goat anti‐rabbit IgG (ab205718, Abcam) at 37°C for 2 h at room temperature.

### Kaplan–Meier analysis

Kaplan–Meier analysis in GBM patients with high or low YTHDF2 expression was employed to assess the prognostic value of YTHDF2 in GBM. They were stratified into two groups: the median of YTHDF2 was taken as the threshold. Those more than or equal to the median were the high YTHDF2 group, and those less than the median were the low YTHDF2 group. Combined with the clinical data, the survival rate with the differentially expressed YTHDF2 was plotted using the ‘survival’ package based on Kaplan–Meier curve analysis.

### Culture and treatment of cells

The GBM cells (T98G and LN229) were obtained from American Type Culture Collection (ATCC, USA) and cultured in DMEM (Gibco, Grand Island, USA) containing 10% FBS (Gibco, USA). For the acquisition of TMZ‐resistant cell lines: TMZ subjection was conducted at (1/50 IC50) in T98G and LN229 cells. The dose of TMZ increased gradually after the stable cell growth. The dose was changed every 15 days till the fifth month. The established TMZ‐resistant cells were named T98G/TR and LN229/TR cells.

The lentivirus and negative controls were purchased from Genepharma. Co., Ltd (Shanghai, China) to infect with the GBM cells. The lentivirus containing different genes or sequences together with the siRNAs, like LV‐YTHDF2 and LV‐EPHB3, LV‐Sh‐YTHDF2 and si‐EPHB3, LV‐YTHDF2 and LV‐TNFAIP3, LV‐sh‐YTHDF2 and si‐TNFAIP3, LV‐sh‐YTHDF2' and si‐EPHB3, and LV‐sh‐YTHDF2' and si‐TNFAIP3 were infected or transfected into T98G and LN229 cells or T98G/TR and LN229/TR cells.

T98G and LN229 cells infected with LV‐YTHDF2 were treated with 10 μM LY294002 (PI3K inhibitor) or 20 μM JSH‐23 (NFκB inhibitor) for 24 h. T98G/TR and LN229/TR cells infected with LV‐sh‐YTHDF2 and transfected with si‐EPHB3 or si‐TNFAIP3 were treated with 10 μM LY294002 or 20 μM JSH‐23 for 24 h. After these treatment, all the cells were subjected to 200 μM TMZ treatment for 48 h.

### Lentiviral infection and cell transfection

The lentivirus containing YTHDF2 (LV‐YTHDF2), negative control LV‐NC, YTHDF2‐2A‐Mut (K416A, R527A) (LV‐YTHDF2‐2A‐Mut), YTHDF2‐3A‐Mut (W432A, W486A, W491A) (LV‐YTHDF2‐3A‐Mut), YTHDF2‐5A‐Mut (K416A, W432A, W486A, W491A, R527A) (LV‐YTHDF2‐5A‐Mut), two independent YTHDF2 shRNAs (LV‐sh‐YTHDF2 and LV‐sh‐YTHDF2′), negative control LV‐sh‐NC, LV‐EPHB3, LV‐TNFAIP3 and siRNA‐mediated knockdown of EPHB3 and TNFAIP3 (si‐EPHB3 and si‐TNFAIP3) was obtained from GenePharma Co., Ltd (Shanghai, China). T98G and LN229 cells (1 × 10^6^ cells/well) were cultured at 37°C with 5% CO_2_. When reaching 80% confluence, the lentivirus and siRNAs were infected or transfected into the cells followed standard guidelines. The medium was replaced by a fresh one after infection for 12 h or transfection for 24 h. For the T98G/TR and LN229/TR cells, they were seeded at the same density at 37°C in the presence of 5% CO_2_, and the remaining procedures were the same as the above.

### CCK8 assay

The CCK8 assay was conducted to assess the viability of GBM cells. T98G and LN229 cells (1 × 10^4^ cells/well), T98G/TR and LN229/TR cells (1 × 10^4^ cells/well) with different treatments were re‐suspended in the DMEM medium and cultured in a 96‐well plate for overnight. Then, a cell counting kit 8 (Beyotime) was applied to measure the cell activity. The CCK8 solution (10 μL) was added and incubated at 37°C for 90 min. Next, the absorbance at 450 nm was recorded on a microplate reader (Infinite M200, Austria).

### EdU staining

The EdU Assay Kit (Click‐iT) was applied to assess the proliferation of T98G, LN229, T98G/TR and LN229/TR cells. The above cells were cultured in a 24‐well plate with 10 μM EdU reagent treatment in 37°C condition for 2 h. Next, the cells were fixed in 4% formaldehyde for 1 h. The EdU‐positive cells were quantified by a fluorescent microscope (Olympus, Japan). The quantitative positive cells were calculated with the Image‐Pro Plus 6.0 software.

### Flow cytometry

The different treatment of T98G, LN229, T98G/TR and LN229/TR cells was collected for staining. According to the standard protocols, the cell apoptosis was determined by an Annexin‐V‐FITC Kit (BD Pharmingen, San Diego, CA, USA). After washing with PBS, Annexin V/propidium iodide was added to cells and incubated for 30 min in the dark, and the apoptotic rate was measured by FACS cytometry (BD Biosciences, USA).

### Tumor xenograft model

A total of 28 nude mice were grouped into LV‐NC + saline, LV‐YTHDF2 + saline, LV‐NC + TMZ and LV‐YTHDF2 + TMZ. Each group was assigned seven mice. Specifically, 5 × 10^6^ infected T98G cells (LV‐NC or LV‐YTHDF2) were injected into the flanks of mice through subcutaneous. When the tumor size grown to 50 mm^3^, each mice received the intraperitoneal injection of 25 mg kg^−1^ saline or 25 mg kg^−1^ TMZ (every two days for 21 days).

In addition, a total of 28 nude mice were allocated into another 4 groups: LV‐sh‐NC + saline, LV‐sh‐YTHDF2 + saline, LV‐sh‐NC + TMZ and LV‐sh‐YTHDF2 + TMZ groups. Each group was assigned seven mice. Specifically, 5 × 10^6^ infected T98G/TR cells (LV‐sh‐NC or LV‐sh‐YTHDF2) and TMZ were injected as the above method. The animal experiments were approved by the Animal Care and Use Committee of Zhengzhou University (2019‐KY‐189).

### Fluorescence *in situ* hybridisation (FISH)

The FISH assay was carried out to confirm the co‐localisation of YTHDF2 and EPHB3 or TNFAIP3 followed the previous description.^51^ The T98G/TR and LN229/TR cells were collected and fixed at 25 ± 2°C for 30 min. Then, RNAscope multiplex fluorescent detection kit was used to conduct the RNA *in situ* hybridisation assay. Followed by the completion of the *in situ* hybridisation, the slides were washed with PBS containing Tween‐20 (PBST) and incubated with the anti‐YTHDF2 (MBL, 1:500) at 4°C overnight. Subsequently, the secondary antibody (Invitrogen, 1:500) was used to the multi‐target fusion protein.

### RNA immunoprecipitation (RIP)

The EZ‐Magna RIP RNA‐binding protein immunoprecipitation kit (Millipore, USA) was used for this assay. Specifically, the cell lysates from T98G, LN229, T98G/TR and LN229/TR cells were incubated with the anti‐IgG (ab190475, Abcam) or anti‐YTHDF2 (ab220163, Abcam)‐conjugated magnetic beads. The RNA in the complex was isolated and the level of EPHB3 or TNFAIP3 was then quantified by qRT‐PCR detection.

### Dual‐luciferase reporter gene assay

The dual‐luciferase reporter gene assay was performed to examine the interaction between YTHDF2 and EPHB3 or TNFAIP3. As previously described,^52^ a luciferase reporter vector including the YTHDF2 binding site of EPHB3‐3'UTR with m^6^A sites (EPHB3‐WT) or the EPHB3‐3'UTR with mutant m^6^A sites (EPHB3‐Mut) was constructed. And the potential YTHDF2 binding sites of TNFAIP3‐3′UTR with m^6^A sites (TNFAIP3‐WT) or the TNFAIP3‐3′UTR with mutant m^6^A sites (TNFAIP3‐Mut) was constructed. Then, the above recombinant vector and sh‐YTHDF2 were co‐transfected into T98G/TR cells or the above recombinant vector and LV‐YTHDF2 were co‐transfected into T98G cells. After 48 h, a dual‐luciferase reporter assay system (Promega, Wisconsin, USA) was used to assess the luciferase activity in each group.

### RNA degradation assay

To understand the impact of YTHDF2 on the mRNA degradation of EPHB3 or TNFAIP3, sh‐YTHDF2 was transfected into T98G/TR and LN229/TR cells followed by treatment of Actinomycin‐D (5 μm) for 0, 3 and 6 h. Next, LV‐YTHDF2 was infected into T98G and LN229 cells followed by the treatment of Actinomycin‐D. Subsequently, the expression of EPHB3 or TNFAIP3 mRNA was detected by qRT‐PCR.

### Immunofluorescence analysis

After culture for 24 h, the T98G, LN229, T98G/TR and LN229/TR cells were fixed in 4% paraformaldehyde and blocked in 5% BSA for 1 h. Next, the antibodies of anti‐YTHDF2 and anti‐EPHB3 (Abcam) were added to cells and incubated at 4°C for overnight. In the following, cells were incubated with secondary antibodies and incubated with DAPI for 10 min. Ultimately, the immunofluorescence result was observed under a fluorescent confocal microscope.

### Statistical analysis

GraphPad Prism 5 (GraphPad Software, CA) was applied to perform all the statistical analyses. Data are expressed as mean ± standard deviation of three independent assays. The Student's *t*‐test or one‐way ANOVA was conducted for comparison between two or multiple groups. The correlation between YTHDF2 and EPHB3 or TNFAIP3 expression in GBM tissues was analysed through Pearson correlation coefficient analysis. *P < *0.05 indicates the statistical difference.

## Conflicts of interest

All authors declare that they have no conflict of interest.

## Author contribution

**Yu Chen:** Conceptualization. **Yan‐lan Wang:** Data curation; Investigation. **Kai Qiu:** Data curation. **Yi‐qiang Cao:** Investigation. **Feng‐jiang Zhang:** Formal analysis. **Hai‐biao Zhao:** Data curation. **Xian‐zhi Liu:** Conceptualization, Project administration.

## Supporting information

Supplementary Material
