# Supplementary material for: YTHDF2 promotes temozolomide resistance in glioblastoma by activation of the Akt and NF‐κB signalling pathways via inhibiting EPHB3 and TNFAIP3
Source: Clin Transl Immunology. 2022 May 9;11(5):e1393. doi: 10.1002/cti2.1393 (PMC9082891; doi:10.1002/cti2.1393)
Supplement: Supplementary file 1 — Supplementary Material [file CTI2-11-e1393-s001.docx]

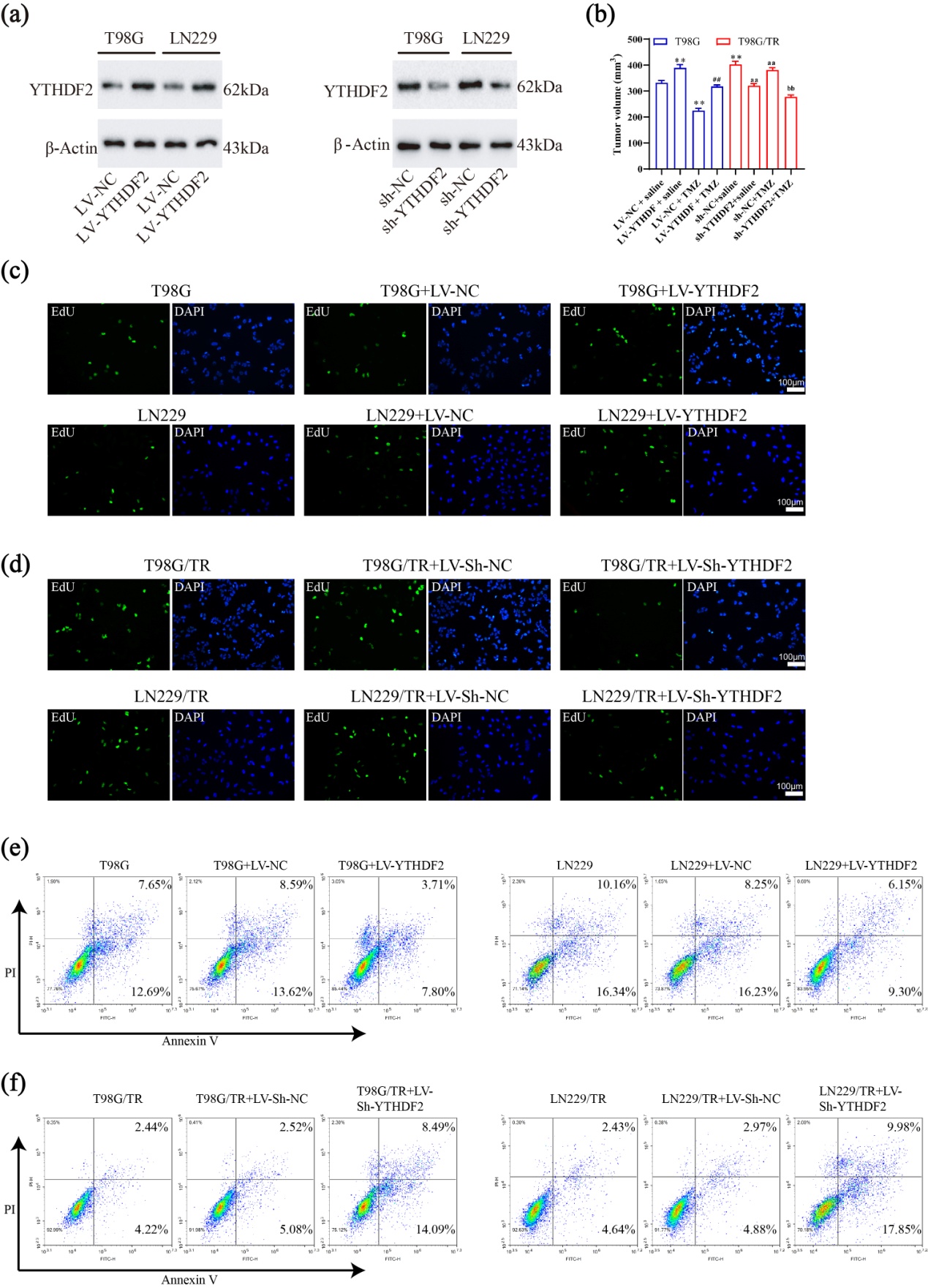


**Supplementary figure 1.** Detection of lentiviral infection efficiency, tumor size, and viability and apoptosis of GBM cells. **(a)** The infections of LV-YTHDF2, LV-NC, LV-sh-YTHDF2 and LV-sh-NC were performed in T98G and LN229 cells, and the YTHDF2 protein level was evaluated. **(b)** T98G cells were infected with LV-YTHDF2, and T98G/TR cells were infected with LV-sh-YTHDF2. Cells were then injected subcutaneously into the ﬂanks of nude mice, followed by intraperitoneally injection of 25 mg kg^-1^ TMZ every 2 days for 21 days. The tumor size was measured after TMZ injection for 21 days. **(c–f)** The LV-YTHDF2 or LV-NC infection on T98G and LN229 cells, and LV-sh-YTHDF2 or LV-sh-NC infection on T98G/TR and LN229/TR cells were performed. EdU assay and flow cytometry assay were carried out to detect proliferation and apoptosis of GBM cells. Data are from three independent experiments. ***P* < 0.01 vs. LV-NC+saline; ##*P* < 0.01 vs. LV-NC+TMZ; aa *P* < 0.01 vs. Sh-NC+saline; bb *P* < 0.01 vs. sh-NC+TMZ; Scale bar: 100 μm.


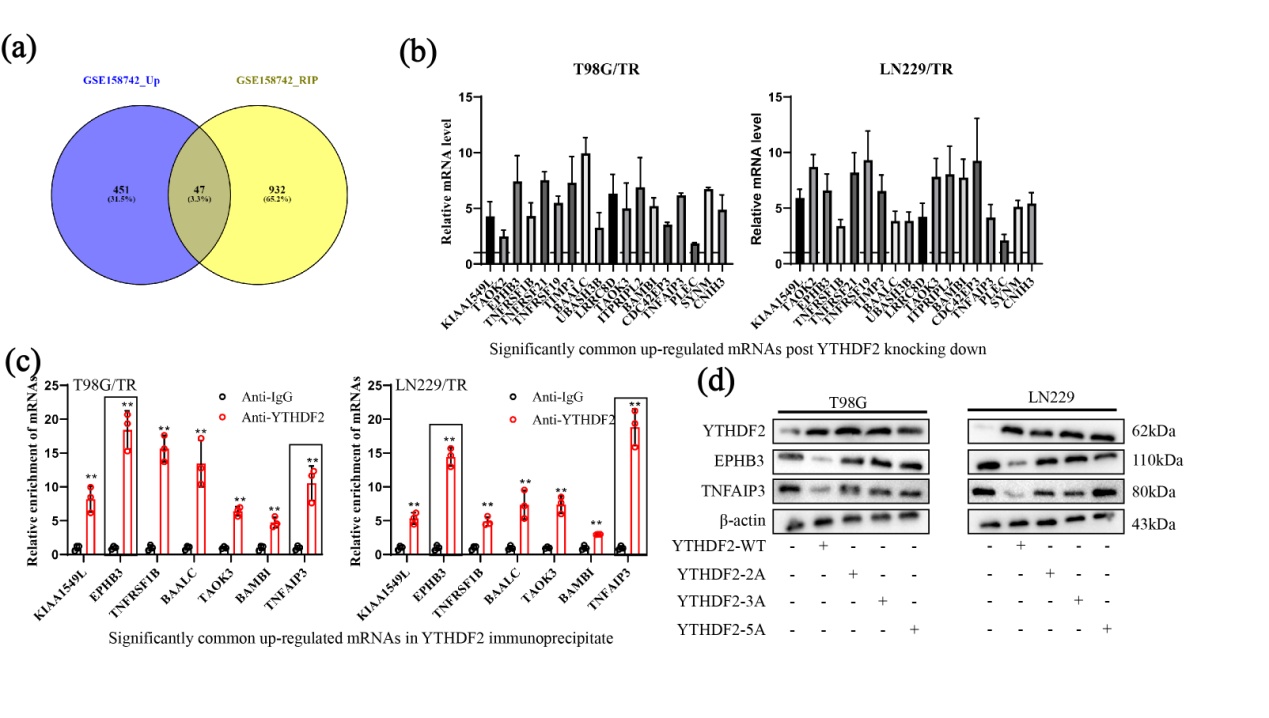


**Supplementary figure 2.** The up-regulated mRNAs were screened and identified after the knockdown of YTHDF2. **(a)** The GSE158742 database was used for the RNA-sequence RIP-sequence analysis, which was based on YTHDF2 silence in stem cell samples from GBM patients. The obtained number of mRNAs was exhibited. **(b)** The LV-sh-YTHDF2 or LV-sh-NC was infected into T98G/TR and LN229/TR cells followed by detection of screened gene mRNA level. **(c)** RNA immunoprecipitation (RIP) was performed using anti-YTHDF2 antibody or anti-IgG in T98G/TR and LN229/TR cells to identify which genes could bind to YTHDF2. **(d)** The YTHDF2-WT and mutants of YTHDF2-2A-Mut (K416A, R527A), YTHDF2-3A-Mut (W432A, W486A, W491A) and YTHDF2-5A-Mut (K416A, W432A, W486A, W491A, R527A) were constructed and transfected into T98G and LN229 cells. The protein levels of YTHDF2, EPHB3, and TNFAIP3 were measured. Data are from three independent experiments. ***P* < 0.01 vs. Anti-IgG.


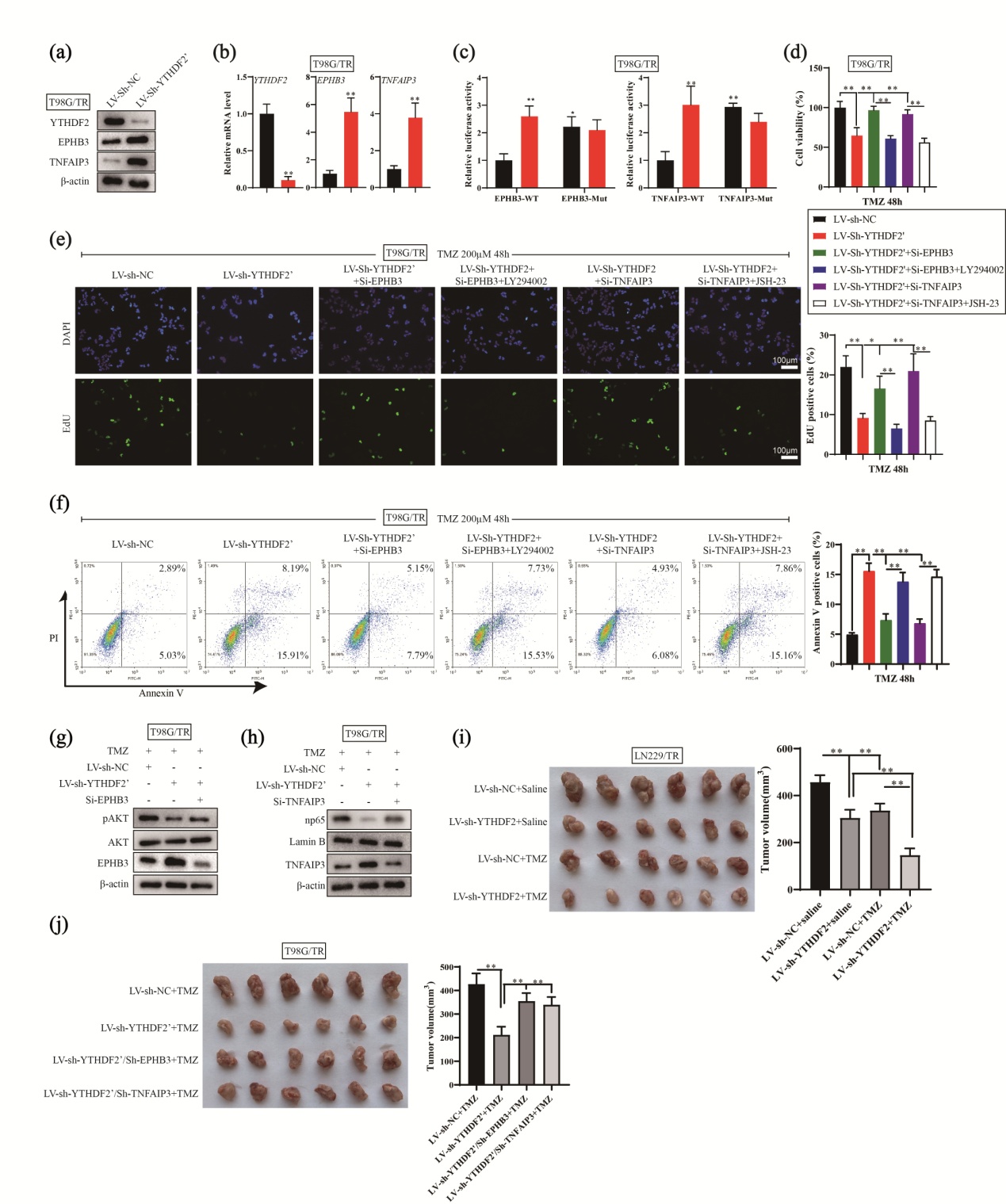


**Supplementary figure 3.** Effect of YTHDF2 silence on the TMZ resistance in GBM cells. **(a, b)** A new shRNA against YTHDF2 carried by lentivirus was employed and named LV-sh-YTHDF2’. The LV-sh-YTHDF2’ or LV-sh-NC was infected into T98G/TR cells, and the protein and mRNA levels were detected. **(c)** The luciferase reporter gene vector EPHB3-3'UTR (EPHB3-WT) or TNFAIP3-3'UTR (TNFAIP3-WT) and EPHB3-3'UTR or TNFAIP3-3'UTR with mutant m6A sites (EPHB3-MUT or TNFAIP3-MUT) were transfected into T98G/TR cells, and the dual-luciferase reporter gene assay was performed to identify the interaction between YTHDF2 and TNFAIP3 or EPHB3. The LV-sh-YTHDF2’ or LV-sh-NC was infected into T98G/TR cells followed by si-EPHB3 or si-TNFAIP3 transfection with or without LY294002 or JSH-23 treatment. The viability **(d)**, proliferation **(e)**, and apoptosis **(f)** of T98G/TR cells were measured. **(g, h)** The protein levels of pAKT, AKT, EPHB3, nuclear p65, Lamin B, and TNFAIP3 in T98G/TR cells were evaluated. **(i)** LN229/TR cells were infected with LV-sh-YTHDF2’ or LV-sh-NC. Cells were then injected subcutaneously into the ﬂanks of nude mice, and the mice were subsequently treated with 25 mg kg^-1^ TMZ by intraperitoneal injection every 2 days for 21 days. The intraperitoneal injection of saline was performed in the mice without TMZ injection (n = 7, per group). The tumor size was measured after TMZ injection every 2 days for 21 days. At day 21, the tumors were collected and photographed. **(j)** T98G/TR cells were infected with LV-sh-YTHDF2’ or LV-sh-NC and transfected with sh-EPHB3 or sh-TNFAIP3. Cells were then injected subcutaneously into mice, and the mice were subsequently treated with TMZ as above (n=7, per group). The tumor size was measured after TMZ injection every 2 days for 21 days. The tumors were photographed and the size was analyzed at day 21. Cell data are from three independent experiments. **P* < 0.05, ***P* < 0.01 vs. LV-sh-NC or LV-sh-YTHDF2’ or LV-sh-YTHDF2’+si-EPHB3 or LV-sh-YTHDF2’+si-TNFAIP3 or LV-sh-NC+saline or LV-sh-NC+TMZ; Scale bar: 100 μm.

**Supplementary table 1. The sequences of primers for qRT-PCR detection.**

| **Gene name** | **Primer sequence (5’-3’)** |
| --- | --- |
| METTL3 | Forward: ACCTATGCTGACCATTACCAAG |
|  | Reverse: CTGTTGGTTCAGAAGGCTCTC |
| METTL14 | Forward: GTTGGA ACATGGATAGCCGC |
|  | Reverse: CAATGCTGTCGGCACTTTCA |
| WTAP | Forward: TTCCCAAGAAGGTTCGATTG |
|  | Reverse: TGCAGACTCCTGCTGTTGTT |
| FTO | Forward: CGGTATCTCGCATCCTCATT |
|  | Reverse: ATTTCAGCCTCGGTGTGTTT |
| ALKBH5 | Forward: GCCTATTCGGGTGTCGGAAC |
|  | Reverse: CTGAGGCCGTATGCAGTGAG |
| YTHDF1 | Forward: GCACACAACCTCCATCTTCG |
|  | Reverse: AACTGGTTCGCCCTCATTGT |
| YTHDF2 | Forward: TCTGGAAAAGGCTAAGCAGG |
|  | Reverse: CTTTTATTTCCCACGACCTTGAC |
| YTHDF3 | Forward: TGACAACAAACCGGTTACCA |
|  | Reverse: TGTTTCTATTTCTCTCCCTACGC |
| PTEN | Forward: CCCACCACAGCTAGAACTTATC |
|  | Reverse: TCGTCCCTTTCCAGCTTTAC |
| β-actin | Forward: CTCCATCCTGGCCTCGCTGT |
|  | Reverse: GCTGTCACCTTCACCGTTCC |

**Supplementary table 2. Correlation between YTHDF2 expression level and clinic pathological characteristics in patients with glioblastoma.**

| **Characteristics** | **All cases**  **n=41** | **YTHDF2** | | |
| --- | --- | --- | --- | --- |
|  |  | **High expression** | **Low expression** | **P-value** |
| Age |  |  |  | 0.2623 |
| <50 | 18 | 11 | 7 |  |
| ≥50 | 23 | 10 | 13 |  |
| Gender |  |  |  | 0.4439 |
| Male | 25 | 14 | 11 |  |
| Female | 16 | 7 | 9 |  |
| Chemotherapy resistant |  |  |  | 0.002 |
| R | 16 | 3 | 13 |  |
| NR | 25 | 18 | 7 |  |

R, the responder; NR, non-responder.
